# Rare Presentation of Atypical Hemolytic Uremic Syndrome in an Adult

**DOI:** 10.7759/cureus.18184

**Published:** 2021-09-22

**Authors:** Ammar Alhabhbeh, Zainab Fatima, Akesh Thomas, Christopher Cook

**Affiliations:** 1 Internal Medicine, East Tennessee State University, Johnson City, USA; 2 Internal Medicine, East Tennessee State University Quillen College of Medicine, Johnson City, USA

**Keywords:** eculizumab, acute renal failure, atypical hemolytic uremic syndrome (ahus), tma, thrombocytopenia, hemolytic anemia, microangiopathic hemolytic anemia, hus

## Abstract

Thrombotic microangiopathies (TMA) are disorders characterized by microangiopathic hemolytic anemia, thrombocytopenia, and microthrombi leading to organ dysfunction. Atypical hemolytic uremic syndrome (aHUS) is a rare subtype of TMA mediated by complement dysregulation. We present a case of a 59-year-old female who presented with acute kidney injury and mild thrombocytopenia but with normal hemoglobin. We highlight the importance of prompt diagnosis of aHUS and initiating appropriate treatment with eculizumab.

## Introduction

Thrombotic microangiopathy (TMA) can be broadly classified into thrombotic thrombocytopenic purpura (TTP) or hemolytic uremic syndrome (HUS), with HUS further classified as typical HUS caused by Shiga toxin-producing *Escherichia coli* infection and atypical HUS (aHUS). aHUS is a rare form of complement-mediated TMA most commonly caused by mutations in the alternative complement cascade, resulting in the overactivation of complements which causes endothelial damage. Endothelial breakdown leads to platelet activation and consumption, microthrombi formation, and erythrocyte lysis, leading to hemolytic anemia and schistocytes [1]. While the pathophysiology of various TMAs is different, clinically, it can be challenging to distinguish aHUS from TTP or Shiga toxin-mediated HUS.

Physicians need to differentiate TTP from aHUS as trials have shown the efficacy of eculizumab in treating aHUS, particularly with regard to improvement in renal function [2].

## Case presentation

Our patient was a 59-year-old female with a past medical history of obesity and type 2 diabetes complicated by neuropathy; her medications include metformin and gabapentin. She initially developed nausea and non-bilious vomiting, along with abdominal pain and diarrhea for two days after eating at a local gathering. She denied any melena, hematemesis, or fever/chills. Abdominal symptoms were followed by altered mental status, confusion, and hallucinations two days later, which prompted the family to bring her to the emergency department (ED). In the ED, she was tachycardic, but other vitals remained stable. Physical examination revealed diffuse abdominal tenderness without rebound or guarding, and the initial neurological examination showed confusion and agitation.

**Figure 1:**
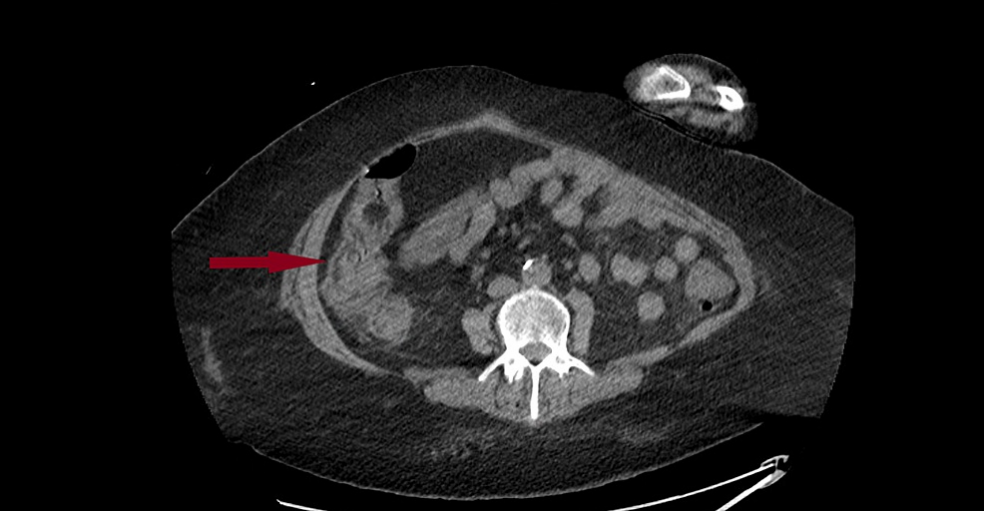
CT abdomen and pelvis without contrast: showing diffuse thickening of colonic wall with surrounding inflammatory changes. CT, computed tomography

Her initial lab work was significant for leukocytosis (15.9 k/uL) with a neutrophilic predominance and mild thrombocytopenia (139 k/uL), but she had normal hemoglobin of 12.9 g/dL. In addition, she had acute kidney injury with a creatinine of 5.75 mg/dL and blood urea nitrogen of 56 mg/dL. At the time of admission, she had mildly elevated transaminases but normal bilirubin. Computed tomography (CT) scan of abdomen and pelvis without contrast revealed diffusely thick-walled colon with surrounding inflammatory change (Figure 1 ).

The findings raise the possibility of pseudomembranous colitis/*Clostridium difficile*. Our initial differential diagnosis was severe gastroenteritis with colitis due to possible *C. difficile* infection leading to volume depletion, acute kidney injury, and metabolic encephalopathy. CT head without contrast was unremarkable. She was started on intravenous fluid and antibiotics, and the nephrology team was consulted to initiate hemodialysis. Unfortunately, her kidney function worsened, and she was started on hemodialysis on the second day of admission. Stool analysis was negative for *C. difficile*, Shiga toxin, and campylobacter. 

Her mental status deteriorated, and she became stuporous, which led to intubation to protect the airway. By the third day of admission, the patient had one session of hemodialysis with a slight improvement in her creatinine. However, she developed worsening anemia with hemoglobin of 9.8 g/dL and thrombocytopenia with a platelet count of 66 k/uL. Peripheral smear obtained was notable for frequent schistocytes suggestive of a microangiopathic hemolytic process. The trend for her labs is shown in Table 1.

**Table 1:** Lab values on day 1 and day 3

Initial Investigation	FirstDay of Admission	Third Day of Admission	Reference Range
Hemoglobin	12.9 g/dL	9.8 g/dL	12.4-15.2 g/dL
Hematocrit	39.1%	29.4%	41%-51%
Platelet	139 k/uL	90 k/uL	150-400 k/uL
White blood cell	15.9 k/uL	9.1 k/uL	3.5-11 k/uL
Creatinine	5.75 mg/dL	7.48 mg/dL	0.5-1.35 mg/dL
Blood urea nitrogen	56 mg/dL	63 mg/dL	6-23 mg/dL
Alanine transaminase	142 U/L	98 u/L	0-53 U/L
Aspartate aminotransferase	162 U/L	73 U/L	0-39 U/L
Alkaline phosphatase	128 U/L	87 U/L	39-117 U/L
Total bilirubin	0.8 mg/dL	0.8 g/dL	0.3-1.2 mg/dL

**Figure 2:**
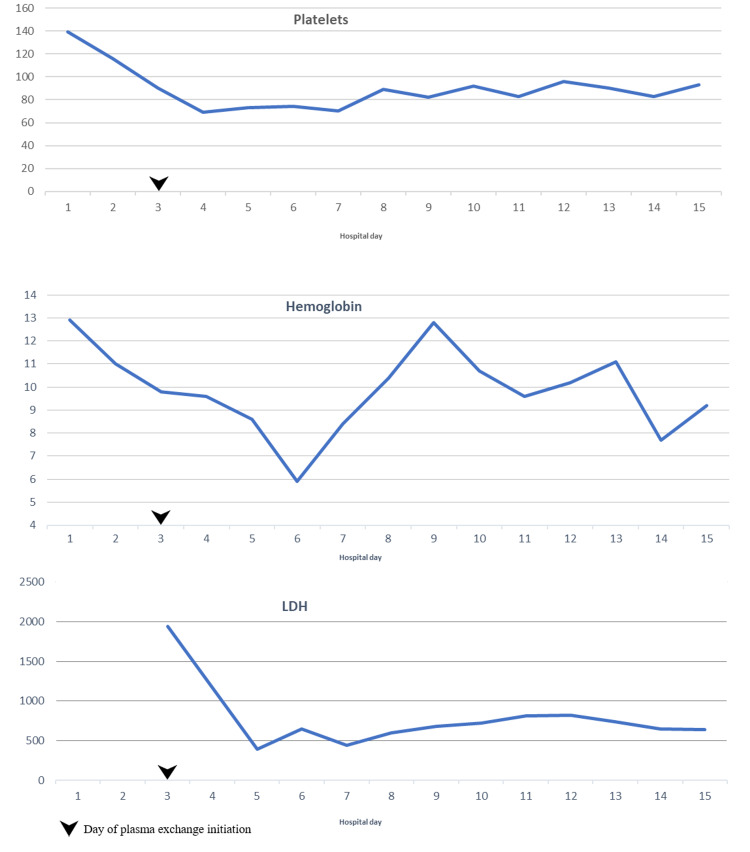
Trend of platelets, hemoglobin, and LDH during the course of admission. LDH, lactate dehydrogenase

Her coagulation studies remained normal, ruling out disseminated intravascular coagulation. Lactate dehydrogenase (LDH) was elevated (1941 U/L), with low haptoglobin (<10 mg/dL) and elevated corrected reticulocyte count; bilirubin was never elevated. We suspected TTP given the microangiopathic hemolytic anemia with schistocyte, thrombocytopenia, altered mental status, and acute renal failure. Plasmapheresis was emergently initiated, and the patient was started on pulse steroids, leading to improvement in thrombocytopenia and eventually her hemoglobin (Figure 2).

She became more responsive with plasma exchange, leading to extubation. Her ADAMTS13 came back as 52% (normal >60%), ruling out TTP. In addition, stool analysis was negative for Shiga toxins ruling out typical HUS. Our patient had a thorough workup for secondary causes of TMA, after which she was diagnosed with aHUS. She was not found to be on any medication that could trigger aHUS. She also had a complete infectious workup which was negative for human immunodeficiency virus (HIV), hepatitis B, or any bloodstream or respiratory tract infections. She also had a complete rheumatological workup which was negative for antineutrophil cytoplasmic antibody vasculitis, systemic lupus erythematosus, or antiphospholipid syndrome. Her complement levels were low with C3 75 mg/dL [83-193 mg/dL], C4 8 mg/dL [15-57 mg/dL], and low total complement (35.4 U/mL) [38.7-89.9 U/mL].

Plasma exchange was discontinued after four cycles. Weekly eculizumab was promptly initiated, and the hemolytic parameters, including anemia, thrombocytopenia, and LDH, improved initially but then declined again (Figure 2), requiring transfusion support. While her mental status improved, she continued to require hemodialysis. The patient was physically deconditioned after a prolonged hospitalization; she refused treatment after four doses of eculizumab and decided to enroll in hospice for comfort care.

## Discussion

TMA describes a group of systemic disorders with characteristic microvascular microthrombi leading to microangiopathic hemolytic anemia and thrombocytopenia [3]. TMA can be classified into primary and secondary types. Primary TMA includes TTP, which is characterized by mutation or low activity of the ADAMTS13 gene (<5-10% activity). HUS is another form of primary TMA, which can further be divided into typical HUS caused by Shiga toxin-producing *E. coli* infection and aHUS due to genetic mutation in complement regulatory proteins [3,4]. Secondary TMA can occur with drugs such as calcineurin inhibitors, pregnancy, malignancy, transplant, autoimmune conditions, and infection such as HIV, Epstein-Barr virus, and parvovirus. It is imperative to rule out secondary causes as treatment involves treating the underlying conditions or discontinuing the offending medication [3]. Our patient’s Shiga toxin was negative, and her ADAMTS13 level was 52%; this led to the investigation of other etiologies of TMA. Other causes such as Streptococcus pneumonia, pregnancy, autoimmune conditions, and offending medications were also ruled out, and she was diagnosed with aHUS.

aHUS is a rare subtype of TMA caused by dysregulation of the complement pathway with an incidence of 0.23-0.43 cases per million population [4]. The complement system, a part of innate immunity, enhances the ability to clear pathogens via classical, lectin, and alternative pathways. In the alternative pathway, C3b deposits on cell surfaces and gets activated to create C3 convertase, which then creates C5 convertase, activating C5 to form membrane attack complex (MAC) and leading to cell death [1,5]. Pathogenesis of aHUS involves excessive activation of the alternative complement cascade; this excessive activation occurs when built-in regulators fail to inactivate the C3b deposit on healthy normal cells. Complement deposit causes endothelial injury, leading to infiltration of leukocytes and platelets, resulting in the formation of obstructive thrombi in the vessels. Microthrombi lead to the shearing of erythrocytes creating schistocytes and microangiopathic hemolytic anemia. Platelet consumption at the site of endothelial injury leads to thrombocytopenia. Renal impairment is due to complement deposition leading to thrombi formation in renal vasculature [1,4].

In aHUS, this failure most commonly occurs due to a loss of function of a regulatory protein either by genetic mutation or autoantibody. Genetic mutation is implicated in 40-60% of aHUS cases; mutations in CFH, CFI, MCP, and CF3 can predispose to aHUS. Although in those predisposed individuals, a trigger is usually needed for aHUS to occur. Most common triggers include infections, autoimmune conditions, drugs, malignancies, or pregnancy [4-6].

aHUS should be suspected in a patient presenting with TMA once Shiga toxin HUS, secondary HUS, and TTP are ruled out. aHUS usually presents with a triad of microangiopathic hemolytic anemia, thrombocytopenia, and renal failure. Cataland et al. found mean platelets of 66 k/uL in patients with aHUS [7]. However, aHUS is associated with profound renal insufficiency requiring hemodialysis compared to TTP [4]. While neurological symptoms are more common in patients with TTP, a minority of patients with aHUS may display neurologic symptoms [8]. Our patient presented with severe renal failure and altered mental status but lacked the typical features of anemia or significant thrombocytopenia at admission, complicating the diagnosis and delaying treatment.

Some patients with aHUS have low C3 levels in blood with normal C4 levels, although the use of complement levels for diagnosis was not found to be adequately sensitive [9]. Our patient had a decrease in both C3 and C4. In a study conducted by Noris et al., only 7% of people with complement gene mutations had low C4 levels [10].

Gene sequencing for complement mutation can also be used to confirm the diagnosis of aHUS but is not required for diagnosis [11]. These mutations include CFH, CFI, CFB, C3, MCP, CDKE, and THBD [4]. However, these tests are expensive, and their results are usually obtained after weeks, thus they cannot be used for clinical decision making. According to a study conducted by Fakhouri et al., 51% of the patients with aHUS had complement gene mutations or autoantibodies [11]. According to the recent literature, the presence or absence of these mutations does not indicate the response to eculizumab therapy [9]. Unfortunately, our patient decided to pursue hospice care before being evaluated for these genetic mutations.

Since the ADAMTS13 test takes several days to process, most patients with suspected TMA are treated with emergent plasma exchange and steroids [3]. While plasma exchange is still the first-line treatment for TMA, including TTP, it is less effective in aHUS than TTP, as it does not address the underlying complement dysfunction [4]. Therefore, in cases of TMA where the diagnosis of aHUS is suspected (normal activity of ADAMS13) and the response to plasma exchange is poor, therapy with eculizumab should be initiated, and plasma exchange should be discontinued [9]. Our patient was also initially treated with plasma exchange, which led to mild improvement in hemolytic markers and thrombocytopenia. However, plasma exchange was discontinued after four cycles once TTP was ruled out, and the patient was initiated on eculizumab.

Eculizumab is a monoclonal IgG antibody that binds to C5 and prevents the formation of the MAC (C5b-9), responsible for the pathogenesis of aHUS [2]. Eculizumab is given an induction dose of 900 mg weekly for four weeks followed by a 1200 mg maintenance dose at week 5 and then 1200 mg every two weeks [9]. In a study by Fakhouri et al. conducted on 41 patients with aHUS receiving eculizumab, 40 patients (98%) had platelet count normalization at a median of eight days. By week 26, 88% of patients had hematologic normalization (normalization of platelet count and LDH), and 54% of patients had estimated glomerular filtration rate improvement of ≥15 mL/min/1.73 m^2^ [11].

The appropriate duration of eculizumab treatment in aHUS has not yet been established [4]. However, an analysis of two trials of eculizumab in aHUS demonstrated that the benefit of eculizumab therapy was maintained at two years follow-up [12]. Furthermore, a recent prospective multicentric study by Fakhouri et al. showed that eculizumab could be safely discontinued in selected patients after aHUS has achieved remission. In patients with no complement gene variants, the risk of aHUS relapse was less than 5% but was significantly higher in carriers of complement gene variants reaching 50%. Time to relapse ranged from 1 to 22 months and was primarily precipitated by infections [13].

## Conclusions

aHUS is a rare disease most commonly caused by a mutation in complement regulatory protein. It can be challenging to diagnose, especially if the anemia and thrombocytopenia are not profound at presentation. There are no clear diagnostic criteria for aHUS, and it remains a diagnosis of exclusion. It is vital to diagnose aHUS promptly as prompt initiation of eculizumab can lower mortality and morbidity. 

## References

[1] Jokiranta TS (2017). HUS and atypical HUS. Blood.

[2] Legendre CM, Licht C, Muus P (2013). Terminal complement inhibitor eculizumab in atypical hemolytic-uremic syndrome. N Engl J Med.

[3] Arnold DM, Patriquin CJ, Nazy I (2017). Thrombotic microangiopathies: a general approach to diagnosis and management. CMAJ.

[4] Raina R, Krishnappa V, Blaha T, Kann T, Hein W, Burke L, Bagga A (2019). Atypical hemolytic-uremic syndrome: an update on pathophysiology, diagnosis, and treatment. Ther Apher Dial.

[5] Sridharan M, Go RS, Willrich MA (2018). Atypical hemolytic uremic syndrome: review of clinical presentation, diagnosis and management. J Immunol Methods.

[6] McFarlane PA, Bitzan M, Broome C (2021). Making the correct diagnosis in thrombotic microangiopathy: a narrative review. Can J Kidney Health Dis.

[7] Cataland SR, Yang S, Wu HM (2012). The use of ADAMTS13 activity, platelet count, and serum creatinine to differentiate acquired thrombotic thrombocytopenic purpura from other thrombotic microangiopathies. Br J Haematol.

[8] Kottke-Marchant K (2017). Diagnostic approach to microangiopathic hemolytic disorders. Int J Lab Hematol.

[9] Cataland SR, Wu HM (2014). Diagnosis and management of complement mediated thrombotic microangiopathies. Blood Rev.

[10] Noris M, Caprioli J, Bresin E (2010). Relative role of genetic complement abnormalities in sporadic and familial aHUS and their impact on clinical phenotype. Clin J Am Soc Nephrol.

[11] Fakhouri F, Hourmant M, Campistol JM (2016). Terminal complement inhibitor eculizumab in adult patients with atypical hemolytic uremic syndrome: a single-arm, open-label trial. Am J Kidney Dis.

[12] Licht C, Greenbaum LA, Muus P (2015). Efficacy and safety of eculizumab in atypical hemolytic uremic syndrome from 2-year extensions of phase 2 studies. Kidney Int.

[13] Fakhouri F, Fila M, Hummel A (2021). Eculizumab discontinuation in children and adults with atypical hemolytic-uremic syndrome: a prospective multicenter study. Blood.

